# Regulation of the length of neuronal primary cilia and its potential effects on signalling

**DOI:** 10.1016/j.tcb.2023.05.005

**Published:** 2023-06-09

**Authors:** Viviana Macarelli, Eleni Leventea, Florian T. Merkle

**Affiliations:** 1 Metabolic Research Laboratories, Wellcome Trust – Medical Research Council Institute of Metabolic Science, University of Cambridge, Cambridge CB2 0QQ, UK; 2 Wellcome Trust – Medical Research Council Cambridge Stem Cell Institute, University of Cambridge, Cambridge CB2 0AW, UK; 3 Wolfson Diabetes and Endocrine Clinic, Cambridge University Hospitals NHS Foundation Trust, Cambridge CB2 0QQ, UK

## Abstract

Primary cilia protrude from most vertebrate cell bodies and act as specialized ‘signalling antennae’ that can substantially lengthen or retract in minutes to hours in response to specific stimuli. Here, we review the conditions and mechanisms responsible for regulating primary cilia length (PCL) in mammalian nonsensory neurons, and propose four models of how they could affect ciliary signalling and alter cell state and suggest experiments to distinguish between them. These models include (i) the passive indicator model, where changes in PCL have no consequence; (ii) the rheostat model, in which a longer cilium enhances signalling; (iii) the local concentration model, where ciliary shortening increases the local protein concentration to facilitate signalling; and (iv) the altered composition model where changes in PCL skew signalling.

## Dynamic changes in the length of primary cilia

**Figure 1:**
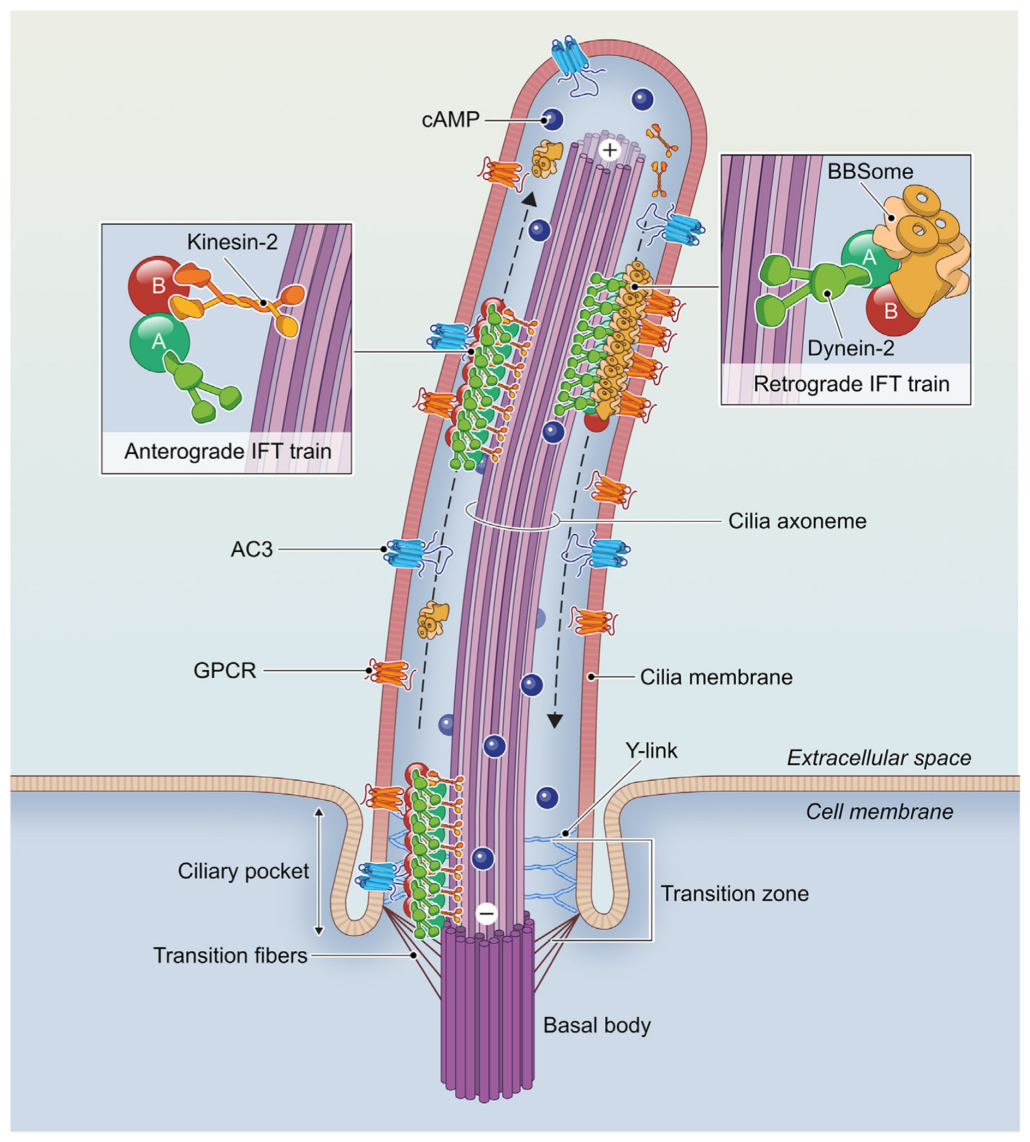
Structure of neuronal primary cilia. The primary cilium projects from the cell body and is built around a microtubule-based axoneme templated from the basal body at the ciliary base. Here, certain membrane proteins engage with adaptor proteins to couple to IFT trains that transport them through the transition zone and along the axoneme toward the cilium’s tip. This active process gives the ciliary membrane a protein and phospholipid composition that is distinct from that of the plasma membrane, indicated by different shading. Abbreviations: AC3, adenylate cyclase 3; cAMP, cyclic AMP; GPCR, G-protein coupled receptor; IFT, intraflagellar transport.

Primary cilia are long, thin, nonmotile organelles that project from the plasma membrane of most vertebrate cell types (Figure 1). At their core is an **axoneme** (see Glossary) of microtubule doublets that arise from a centriole-derived basal body, which anchors to the plasma membrane during the cell cycle’s interphase [1]. The axoneme is enclosed in a lipid bilayer that differs from the plasma membrane in both its lipid and protein content, making the cilium a privileged cellular compartment with a distinct membrane and matrix composition [2]. The distinctive composition of the cilium is maintained by a **transition zone** complex, a specialized domain at the ciliary base [3] that prevents the free diffusion of large soluble proteins (= 100 kDa) and vesicles containing membrane-associated proteins that require specific import mechanisms [2]. Once at the base of the cilium, an encoded ciliary localisation sequence enables these membrane proteins to couple to the **intraflagellar transport (IFT)** machinery aided by the tubby family proteins TUB and TULP3 [4–6]. The IFT proteins form two main complexes, IFT-A and IFT-B, which assemble into long polymeric structures known as ‘trains’ coupled to Kinesin-2 and Dynein-2 motor proteins and their cargoes (Figure 1). IFT-B associated Kinesin-2 moves the train in the anterograde direction toward the ciliary tip, where molecular rearrangements allow the activation of Dynein-2 and the inhibition of Kinesin-2, resulting in retrograde transport back to the ciliary base with the help of the **BBSome** [2,7].IFT plays a central role in ciliogenesis and maintenance of the cilium’s composition, including the composition of the membrane proteins involved in signalling [2]. Indeed, as discussed below, primary cilia play a crucial role in cell signalling, since genetic mutations that disrupt the function of primary cilia in the brain can have profound effects on the development, physiology, and behaviour of the organism, and can lead to a class of diseases collectively referred to as **primary ciliopathies** [8].

Here, we consider another mechanism that may affect ciliary signalling: dynamic alterations in **PCL**. The length of primary cilia can vary over a wide range across different cell types, organisms, and stages of the cell cycle, since cilia are disassembled during cell division and then later reassembled, as reviewed elsewhere [1]. For clarity, here, we consider the mechanisms that affect the PCL in postmitotic **nonsensory neurons** of the healthy mammalian central nervous system, in which primary cilia are relatively stably established, and where the signalling role of primary cilia is likely to contribute to normal brain function [9]. We further define changes in PCL as alterations in the mean PCL of a given cell population, since the absolute length varies within a given cell type. We do not consider mutations that significantly perturb the cilium’s structure or function, but instead focus on the responses of primary cilia to physiological changes. Finally, although changes in PCL are also associated with aging [10] and pathological conditions such as Alzheimer’s disease [11] and Parkinson’s disease [12], the long timeframe of these conditions complicates the discovery of causal relationships, so we focus here on the changes in PCL that occur within the timeframe of minutes to hours. We propose that these stimulus-induced changes in PCL in mammalian neurons may alter the concentration and composition ofciliary of ciliary membrane proteins to affect the function of primary cilia at the level of an organelle, which, in turn, could affect cellular behaviour, ultimately leading to behavioural and/or physiological changes at the level of the organism.

In mammalian neurons, primary cilia range in length from approximately 2 μm[13–16] to12μm [11, 12, 17]. Primary cilia conservatively have 1 00-fold less surface area and 2500-fold less volume than a cell body, assuming a soma 10 μm in diameter and a primary cilium 5 μm in length and 0.2 μm in diameter. In reality, these differences are likely to be much more pronounced since a neuron’s axon and dendrites contain the overwhelming majority of its volume and surface area. Thus, ciliary localisation enables a substantially higher local concentration of ciliary membrane proteins to facilitate efficient signal detection and transduction, leading to the widespread recognition of primary specialized signalling hub or ‘sensory antenna’ [3]. Indeed, primary cilia play a pivotal role in olfactory sensory neurons, which have multiple cilia that are highly enriched in odor-ant-ant-sensitive G-protein coupled receptors (GPCRs) [3,18], and photoreceptor neurons in which the outer segments are specialized primary cilia that house the light-sensitive GPCR rhodopsin [19]. They are also important mediators of numerous signalling pathways including the Sonic Hedgehog (SHH), Wingless-related integration site (WNT), and Transforming Growth Factor β and Bone Morphogenic Protein (TGFβ/BMP) pathways, and thereby broadly affect cell specification, tissue development, and tissue homeostasis [18].

The essential role of primary cilia in mediating signal transduction during development is clearly demonstrated by the severe defects that can emerge when cilia are functionally perturbed in primary ciliopathies [8]. These diseases can arise from disruptions in the cilia’s structure, as seen with the loss of IFT proteins [8], and from disruptions in the cilia’s function, for example, due to an altered ciliary protein composition, as seen with mutations of the genes associated with Bardet–Biedl syndrome (BBS) [8] that perturb the normal ciliary localisation of GPCRs, including SSTR3, MCHR1, and NPY2R [6,20]. Each of these GPCRs is involved in regulating food intake and body weight, suggesting that their disrupted signalling may contribute to the hyperphagia and obesity in patients with BBS mutations. In addition to providing a static environment where receptors, transducers, and second messengers are brought close together, primary cilia can also mediate signalling by dynamically changing the localisation of ciliary proteins. For example, SHH signalling is regulated by the trafficking of the receptors Patched 1 (PTCH1) and GPR161 out of the cilium upon ligand binding, and the ciliary entry of Smoothened (SMO), which then activates the downstream mediators [21].

## Physiological drivers of changes in neuronal PCL

**Figure 2:**
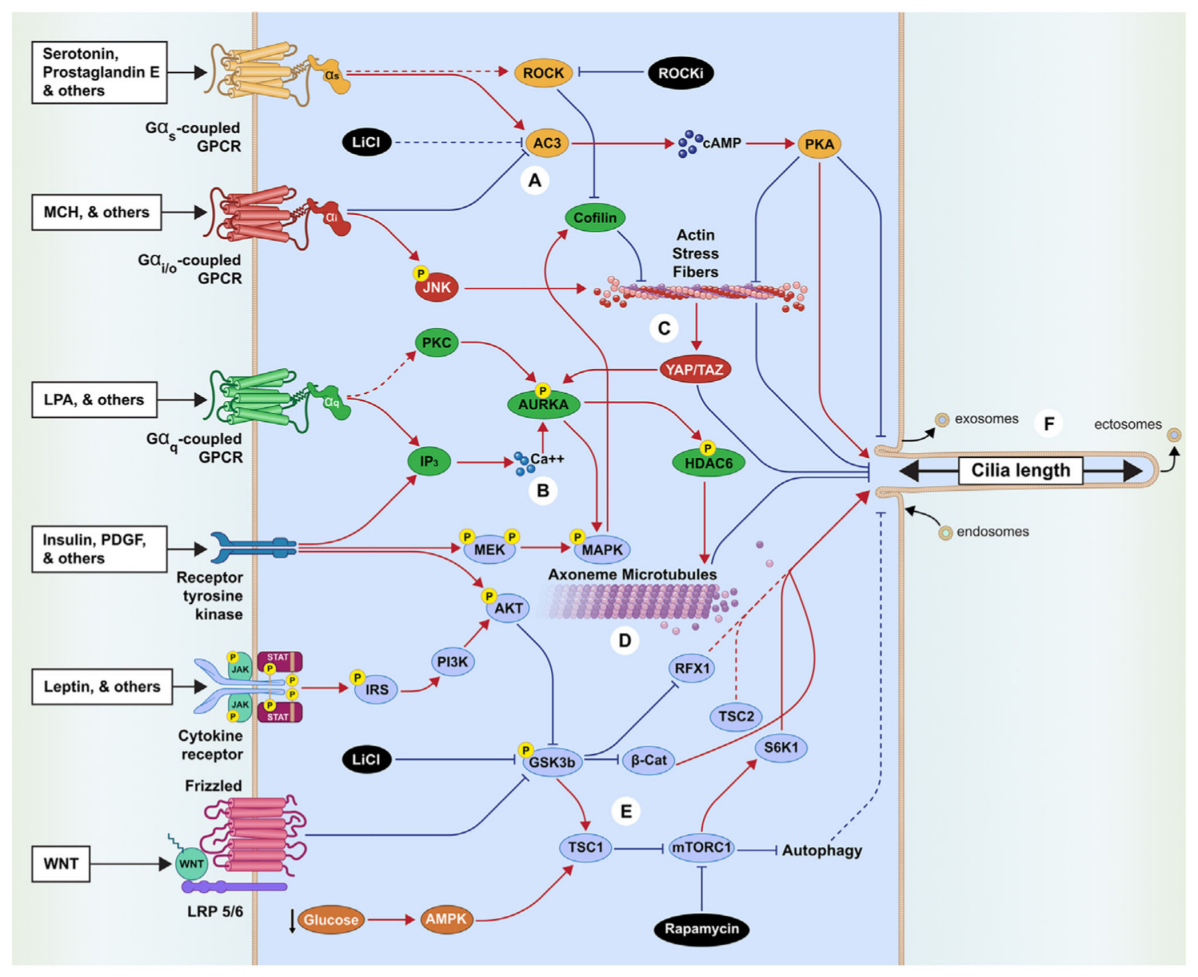
Overview of the signalling pathways controlling changes in PCL. This schematic represents a generic cell, showing the receptors at the plasma membrane (or primary cilium) to the left, their downstream signalling pathways within the cell (light blue), and their effects on PCL to the right. The involvement of each indicated pathway may differ depending on the cell type or cellular state, and are explained in detail in the main text. Critical signalling components are indicated in coloured ovals and are coloured by pathway. Black ovals indicate drugs acting on these pathways, yellow circles with a central ‘P’ indicate protein phosphorylation, and dotted lines indicate controversial or unclear evidence. Abbreviations: AC3, adenyate cyclase 3; GPCR, G-protein coupled receptor; IP3, inositol 1,4,5- trisphosphate; JNK, Jun N-terminal kinase; LiCl, lithium chloride; LPA, lysophosphatidic acid; MCH, melanin concentrating hormone; MEK, mitogen-activated protein kinase kinase; PCL, primary cilia length; PDGF, platelet-derived growth factor; PI3K, phosphatidylinositol 3-kinase; PKA, protein kinase A; RFX1, regulatory factor X; S6K1, ribosomal protein S6kinase 1; WNT,Wingless-related integration site.

Physiological drivers of changes in neuronal PCL Neuronal primary cilia can substantially shrink or grow in response to either physiological or pathological processes [11,22], and exhibit significant changes in length within hours [23,24]. For example, in the hippocampal neurons of mice, acutely administered serotonin increases PCL, partly through activation of the ciliary seroton inreceptor HTR6[11]. Indeed,the over expressio no fHTR6 elongates cilia in the cortical neurons of mice [17], while HTR6 antagonists reduce the length of cilia in the primary striatal neurons of mice [23]. Similarly, agonists of the Gα_s_-coupled ciliary dopamine receptor DRD1 induce elongation of cilia [25], whereas agonists of the Gα_i_-coupled ciliary DRD2 cause shortening of the cilia [12], suggesting these divergent effects are tied to ciliary production of cyclic adenosine monophosphate (cAMP) [26,27] (Figure 2A).

Alterations in neuronal PCL are also seen in response to diverse physiological changes. Some of the most compelling evidence comes from studies of the hypothalamus, a brain structure containing neuron populations that regulate many fundamental homeostatic and behavioural processes. For example, a recent preprint suggested that neurons in the suprachiasmatic nucleus(SCN) of mice, which help regulate circadian rhythms, underwent cyclic changes in PCL, ranging in length from ~1 μm at the onset of the dark cycle to ~6 μm at the onset of the light cycle. The same group also suggested that the rhythmicity of these changes in PCL was disrupted by mutations in the core circadian clock genes, and perturbations in the cilia, in turn, disrupted the robustness of suprachiasmatic nucleus-generated circadian rhythms [28]. Although the functional consequences for the cell’s circuitry are still unclear, metabolic signals can also induce significant changes in PCL in hypothalamic neuron populations that regulate food intake [29,30]. Many GPCRs that respond to metabolic signals are localized to the cilium [30], and acute fasting is associated with shorter hypothalamic cilia, whereas cilia lengthen upon refeeding [14]. However, prolonged exposure to a calorie-dense diet is associated with significantly shorter hypothalamic cilia [14]. At the molecular level, PCL is affected by exposure to dietary palmitic acid, the adipocyte-derived hormone leptin, the pancreatic hormone insulin [30,31], and the orexigenic (appetite-promoting) neuropeptide MCH [16]. Specifically, the acute administration of leptin leads to a 50% increase in PCL in leptin-sensitive regions of the hypothalamus, whereas the loss of leptin or its receptor is associated with significantly shorter primary cilia [14]. Similarly, acute insulin treatment increases the length of the cilia in hypothalamic neurons by up to 25%, up to 25%, probably through activation of the phosphoinositide 3 kinase (PI3K) pathway [14] (Figure 2). Glucose deprivation plays a dual role in regulating PCL by both inducing the formation of cilia to increase the fraction of ciliated cells and, at the same time, inducing ciliary shortening via a mechanism that may involve the activation of AMP-activated protein kinase (AMPK) [32] (Figure 2E). Furthermore, MCHR1 localizes to primary cilia in some appetite-regulatory hypothalamic neurons, where its activation reduces PCL in mice [16,24], potentially by inhibiting production of cAMP [33,34] and promoting **actin stress** fibre production [16] (Figure 2A). More broadly, hypothalamic PCL is affected by posttranslation modifications that reflect the organism's metabolic state such as the O-linked β-*N*-acetylglucosamine (O-GlcNAc) modification of serine and threonine residues. Higher levels of this widespread modification reflect higher internal cellular nutrient levels [35], and inhibition of O-GlcNAcylation is associated with elongated cilia [36]. Indeed, O-GlcNAcylation of ciliary α-tubulin and HDAC6 induces shortening of the cilia, probably via disassembly of the ciliary microtubules [36] (Figure 2D). Below, we explore the mechanisms affecting neuronal PCL in greater detail.

## Molecular mechanisms affecting neuronal PCL

### G-protein coupled receptors

The activation of GPCRs and the subsequent changes in the levels of secondary messengers has been shown to affect PCL. For example, ciliary Gα_s_-coupled GPCRs such as EP4 [3] and HTR6 [23] can stimulate the production of cAMP, thus promoting elongation of cilia [37], partly by redistributing actin stress fibres [33], whereas the reduction of cAMP levels by Gα_i_-coupled GPCRs such as DRD2 [12] and MCHR1 [24] promotes shortening of cilia (Figure 2A). By contrast, increases in the somatic concentration of cAMP can decrease PCL[38]. Lithium chloride promotes the growth of PCL and inhibits **adenylate cyclase 3 (AC3)** [39] but is also likely to act on other signalling pathways [40] Figure2A). Similarly, increased ciliaryCa^2+^levels (Figure 2B) activatecal-cium and/or calmodulin (CaM) kinase upstream from Aurora Akinase (AURKA) [41,42] and are associated with reduced PCL [37]. However, calcium-dependent protein kinase (PKC) can also increase PCL via MAPK [43], suggesting that the effects of calcium on PCL depend on its concentration, dynamics, and cellular location, and the cell's state [33].

#### Actin stress fibres and Rho-associated kinases

Actin stress fibres are likely to regulate PCL, since their destabilisation by pharmacological[44,45] or genetic [46] means promotes longer primary cilia (Figure 2C). Mechanistically, CDK10/CycM phosphorylates the RhoA-associated kinase PKN2, which activates **Rho-associated kinase** (ROCK) to promote the expansion of actin stress fibres [46], which, in turn, induces the nuclear translocation of Yes-associated protein/transcriptional coactivator with PDZ-binding motif (YAP/TAZ) [47] to promote the transcription of AURKA and other negative regulators of PCL(Figure 2B) [44,45]. Similarly, the loss of polycystin-1(PC-1)-mediated repression of ROCK signalling leads to increased actin stress fibres and shortening of cilia through the LIMK–cofilin pathway [48], whereas pharmacological inhibition of ROCK by the small molecule Y-27362 promotes the growth of PCL [44, 48](Figure 2C). Finally, cofilin activation by MAPK promotes the inhibition of stress fibres formation [49] and increases PCL [43](Figure 2C).

#### Tubulin and the IFT machinery

Tubulin and the IFT machinery The microtubules of the axoneme are stable compared with cytoplasmic microtubules [50], and they are most dynamic at the ciliary tip, where they can be readily polymerized or depolymerized (Figure 2D). Increased levels of soluble tubulin, which forms the building blocks of the axoneme, lead to increased cilia length, while stabilisation of the microtubule with the drug taxol leads to shortened or absent cilia [51]. Microtubule stabilisation reduces the pool of soluble tubulin and, therefore, may erode the distal tip of the axoneme [51]. The posttranslational modification of tubulin by polyglutamylation and detyrosination affects the motor function of Kinesin-1 and -2, which are required to transport tubulin inside the cilium and may therefore contribute to the regulation of PCL by promoting elongation [52–54], since increasing the activity of anterograde IFT separately increases PCL [37]. Indeed, it has been proposed that PCL might be regulated by the amount and frequency of cargos transported by the IFT into the cilium [55]. Finally, the histone deacetylase HDAC6 acts downstream from AURKA to deacetylate ciliary microtubules and to promote disassembly of the axoneme and reduction in PCL [36] (Figure 2D).

#### Cell cycle-associated proteins

Since the ciliary basal body must detach from the plasma membrane to organize the microtubule apparatus during mitosis, ciliogenesis and the cell cycle are tightly connected [1]. Postmitotic neurons continue to express cyclins and other proteins typically associated with cell cycle regulation, suggesting that they continue to play a role in regulating PCL. For example, the master cell cycle regulatory complex APC–Cdc20 is required for the normal morphogenesis of dendrites [56]. Cyclin E negatively regulates CDK5 in postmitotic neurons [57] and has been associated with reduced PCL in other cell types [58]. Furthermore, accumulation of the centriolar E3 ligase FBXO41 promotes disassembly of cilia in both mitotic and postmitotic cells in a manner that requires cytoskeletal rearrangements of actin [15].

#### Autophagy and mTOR

Autophagy may regulate PCL, since acute serum starvation is a common experimental tool to induce ciliation *in vitro* [59]. By contrast, sustained serum starvation has been shown to downreg-ulate IFT20 and reduce PCL [59] (Figure 2E), whereas the sustained inhibition of autophagy with 3-methyladenine [60] or through disruption of the positive regulators of autophagy (ATG5 and RPGRIP1L) is associated with increased PCL [60]. While serum starvation arrests cell division, and ciliogenesis is coupled to the cell cycle [61], autophagy may be independent of the cell cycle since its chemical or genetic inhibition in hypothalamic neurons is sufficient to reduce PCL [31] (Figure 2E). Furthermore, diseases associated with defects in autophagy, such as focal cortical dyslamination and thyroid Hürthle cell tumours, are characterized by an abnormal PCL [62]. Activation of the autophagy-related serine/threonine protein kinase mTORC1 increases PCL by promoting protein synthesis related to primary cilia assembly [32,63], and its inhibition by the drug rapamycin [64] or by Tuberous Sclerosis Complex 1 (TSC1) leads to reduction in PCL [63] (Figure 2E). Indeed, mTORC1’s activity is downregulated under nutrient-deprived conditions through a signalling cascade involving the activation of AMP-activated protein kinase (AMPK) and TSC1, which promote elongation of cilia when ablated [32] (Figure 2E). The mTOR pathway is also the target of Glycogen synthase kinase-3 beta (GSK3β), which is inhibited by phosphoinositide 3 kinase (PI3K)/AKT signalling downstream from diverse ligands, including the metabolic hormones leptin and insulin [14], which are associated with regulating PCL [65]. Specifically, the inhibition of GSK3β increases β-catenin stability and leads to the growth of PCL [66,67], potentially explaining the effects of lithium chloride on elongation of cilia [40,68]. Inhibition of GSK3β also allows the transcription factor RFX1 to promote anterograde IFT gene expression [66] and relieves the repression of mTORC1 by TSC1 [64] (Figure 2E).

#### Ciliary membrane vesicles

The cilium's length may also be regulated by the incorporation or shedding of membrane-bound vesicles by exocytosis, endocytosis, or ectocytosis. Ciliary exosomes resulting from the exocytosis of multivesicular bodies (MVBs) are thought to be released from the base of cilia where endosomes can also be absorbed, whereas **ectosomes** are produced from the ciliary membrane at the axonemal tip [2,68–71] (Figure 2F). It is unclear whether these processes operate in concert to homeostatically maintain the protein content of the cilium to optimize signalling, or whether they can be engaged independently to alter PCL. Ectosomes contain a fivefold higher concentration of GPCRs than the ciliary membrane [72] and may act as a ‘safety valve’ to rapidly eliminate activated ciliary GPCRs when their retrieval via retrograde IFT fails or is too slow [72]. Although ciliary shedding of ectosomes was first appreciated in cells with defective GPCR retrieval, this phenomenon appears to be active in normal primary cilia [72] and may therefore actively reduce PCL by removing portions of the ciliary membrane. For example, prominin-1-enriched cilia-derived membrane vesicles are associated with shorter primary cilia in neuroepithelial cells, suggesting that their shedding is a mechanism of regulating PCL [73], and cilia from *bbs8* mutant neurons in *Caenorhabditis elegans* display variable length in a manner potentially related to altered ectocytosis [74].

### Potential consequences of changes in PCL

**Figure 3:**
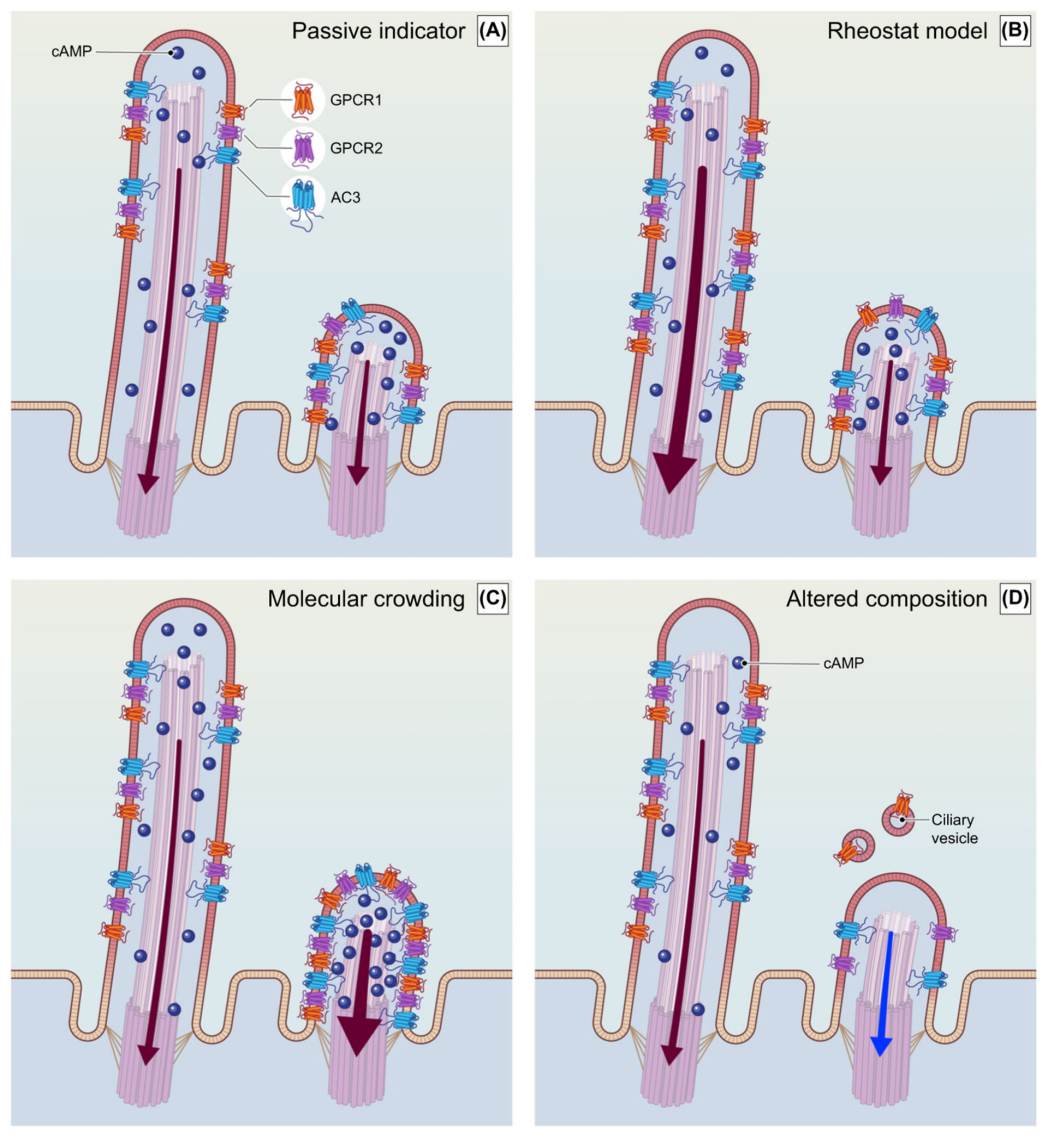
Hypothetical models of the functional consequences of changes in PCL. While many different receptor types and signalling pathways are active in primary cilia, for clarity we schematise ciliary signalling as the interaction of GPCR with AC3 and the downstream production of cAMP as a secondary messenger, with the thickness of the arrow representing the efficacy of signal transduction. (A) The passive indicator model proposes that changes in PCL do not alter ciliary signalling capacity. (B) In the rheostat model, increased PCL potentiates ciliary signalling capacity due to the increased number of ciliary proteins. (C) The local concentration model proposes that if the total number of ciliary proteins is not substantially changed, a shorter cilium would increase their concentration to potentiate or alter ciliary signalling. (D) In the altered composition model, PCL influences the composition and variety of receptors to alter the signalling capacity of the primary cilium. Abbreviations: AC3, adenylate cyclase 3; cAMP, cyclic AMP; GPCR, G-protein coupled receptor; PCL, primary cilia length.

While changes in PCL have been observed in mammalian neurons in response to physiological signals, the consequences of these changes have largely been unexplored. We propose that if the primary cilium acts as a sensory antenna [3,18], changes in the length of this antenna could affect its function as an organelle by modifying its ability to sense and transduce extracellular signals, which, in turn, could affect cellular function and tissue- or organism-level behaviour. Below, we propose four distinct but not mutually exclusive models (Figure 3) of the potential functional consequences of changes in PCL. We expect those to vary by ligand, developmental time point, and cell type.

#### (i) Passive indicator model

Although the primary cilium plays an important role in signalling [18], changes in its length may simply indicate a change in the cell’s state or an echo of past ciliary signalling that does not alter its current or future signalling capacity (Figure 3A). For example, ectosome shedding from the ciliary tip to fine-tune receptor signalling might lead to a reduction in PCL only as a secondary consequence [72]. Furthermore, changes in PCL induced by MAPK, PP-1, and cofilin were not accompanied by obvious changes in ciliary function, as determined by measuring the concentration of cytosolic calcium induced by fluid-shear stress in endothelial cells [43], although we cannot exclude the possibility that functional changes could have been detected with a suitably relevant and sensitive assay. Similarly, experimental manipulations that increased steady-state PCL *in vivo* did not interfere with normal development or behaviour in mice [68,75].

#### (ii) Rheostat model

Alonger ‘sensory antenna’ with a correspondingly larger surface area could increase the number of receptors and downstream signalling mediators without altering their concentration (Figure 3B), so that changes in PCL alter the cilia’s sensitivity to signals, much like a rheostat (variable resistor) regulates the flow of current in an electrical circuit. The rheostat model assumes that the mechanisms regulating proteins’ entry and exit from the primary cilium operate at least as quickly as the observed changes in PCL. This concept is supported by computational models suggesting that the capture rate of molecules by the ciliary receptors scales linearly with PCL [76]. Indeed, the exogenous expression of ciliary proteins has been associated with elongation of cilia in the neocortical neurons of mice [17], suggesting that PCL may change in response to changes in the receptors’ entry or activity to normalize their concentration. We note that despite the constant concentration of receptors and downstream targets (e.g., AC3) imagined in the rheostat model, the concentration of secondary messengers (e.g., cAMP) may still build up to higher levels in the small volume of a longer cilium before they reach the ‘sink’ of the cytoplasm. Experimentally, ciliary SHH signalling was potentiated in mouse embryonic fibroblasts with primary cilia that were lengthened either genetically by the ablation of *Cnsk2α* [77], or pharmacologically by the inhibitor of actin polymerisation Cytochalasin B [78]. In the hypothalamus, alterations in PCL might modulate the intensity of signalling from metabolic factors [30]since leptin- and insulin-induced cilia elongation has been proposed to increase neuronal sensitivity to other metabolic signals [14], leading to increased activation of anorexigenic proopiomelanocortin (POMC) neurons and decreased food intake [22].

#### (iii) Local concentration model

There are a number of ways in which the local concentration of ciliary proteins could be altered. For example, if the rate of protein trafficking into and out of the cilium is slow relative to the rate of change in PCL, then the total number of ciliary membrane proteins would remain relatively constant, increasing their concentration and potentially increasing the number of intermolecular interactions as cilia shrink. This increased local concentration of ciliary proteins could potentiate or alter their signalling in at least three ways: (i) increasing the likelihood that receptors such as GPCRs will interact with downstream targets, (ii) increasing the concentration of second messengers due to the decreased ciliary volume, and (iii) increasing the formation of receptor homomers, heteromers, and higher-order oligomers which could, in turn, shape their responses to ligands [79]. Indeed, longer primary cilia in senescent fibroblasts have been suggested to reduce their capacity to respond to SHH by diluting their receptors and downstream targets [80]. Genetic or pharmacological induction of multiple cilia in the same cell diluted the ciliary proteins and attenuated ciliary signalling, since the multiple primary cilia occupy the same ciliary pocket, and thus the trafficking machinery becomes rate-limiting [81]. Furthermore, the appetite-regulating ciliary GPCRs MCHR1 and SSTR3 heterodimerize [79], and the receptor for the appetite-promoting hormone ghrelin (GSHR) heterodimerizes with multiple other GPCRs including the melanocortin 3 receptor (MC3R) [82], suggesting that changes in PCL that affect local protein concentration could alter neuronal responses to metabolic signals. While it is unclear how ciliary receptors interact with each other within the cilium, they may do so in lipid rafts, which can accumulate at the base of the cilium and have been shown to propagate adipogenic insulin signalling after IR/IGF1R heterodimerization [83].

#### (iv) Altered composition model

Mechanisms that might alter PCL such as ectosome shedding could alter the composition of ciliary proteins and lipids to influence the subsequent signalling, as observed when GPR161 was removed upon SHH binding [72]. The ciliary composition could also be changed by other mechanisms, for example, if longer cilia more effectively accommodate lowly expressed or inefficiently targeted proteins than shorter cilia, in which case ciliary elongation would lead to the presentation of a greater diversity of ciliary proteins.

## Concluding remarks

We have summarized the mechanisms potentially involved in the control of PCL in mammalian neurons and proposed models of how these changes could affect ciliary signalling and, consequently, alter the cell’s state. Here, we propose a set of experiments to determine which model (or combination of models) might explain how PCL could affect signalling pathways, keeping in mind that results from one experimental system may not apply universally (see Outstanding questions). For example, one could use cilia-targeted cAMP or calcium sensors [84] to test how experimentally shortening or lengthening primary cilia might alter ciliary signalling in response to a panel of ligands that act via ciliary GPCRs. One could also use techniques such as somatic calcium imaging or analyses of transcriptional changes to test how changes in PCL might alter the cells’ state. One challenge to this approach is that manipulations that affect PCL may also affect the function of cilia or other compartments, so it would be necessary to validate the findings from one approach using an orthogonal method. Ciliary proximity proteomics could be used to experimentally test how physiologically induced changes in PCL alter ciliary protein composition [85,86]. These experiments would provide rich datasets, but are costly, relatively low-throughput, and have yet to be optimized for neurons. We also note that the molecular mechanisms and models described above have largely been informed by studies in postmitotic mammalian neurons, in which cilia may change in length but otherwise remain relatively stable. However, we propose that the mechanisms and consequences of changes in PCL may be more broadly relevant, since primary cilia in most cell types participate in signalling. In embryonic development, changes in PCL might tune cellular responsiveness, and shape the timing and outcomes of differentiation and tissue morphogenesis. For example, FGF signalling affects the length and function of cilia during development, and developmental defects related to FGF signalling could partly derive from ciliary dysfunction [87]. Changes in PCL are likely to be relevant to disease biology, since the rescue of cilia length attenuates disease phenotypes and mitigates SHH signalling defects [88, 89]. Finally,alterations in PCL are a cellular phenotype that could provide a foundation for image-based clustered regularly interspaced short palindromic repeats (CRISPR) screens, in which the PCL in a given cell is correlated with the identity of the perturbed gene [90]. Such studies could catalogue the genes involved in ciliary formation, function, and length control, as well as revealing a ciliary role for genes associated with human disease. Understanding the causes and consequences of changes in PCL could significantly advance our understanding of primary cilia in development and in the function of adult cells and tissues, and perhaps may also reveal a new class of therapeutic targets for diseases that involve signalling pathways mediated by primary cilia.

## Highlights

Most vertebrate cells, including mammalian postmitotic neurons, have fingerlike primary cilia that protrude from the cell body and act as specialized ‘signalling antennae’ enriched with specific receptors.

Primary cilia length (PCL) can substantially change within minutes to hours in response to physiological changes such as circadian rhythms and metabolic state, and secreted factors such as neurotransmitters and hormones.

The functional consequences of changes in PCL are still poorly understood, but are likely to affect cellular responses to exogenous signals across many cell types and processes.

## Glossary

Actin stress fibres: parallel bundles of actin filaments found in nonmuscle cells that are anchored at the cell membrane, and provide mechanical support and contractile force to cells. The formation of stress fibres is regulated by the Rho family of GTPases.

Adenylate cyclase 3 (AC3): an enzyme that catalyses the conversion of ATP to cyclic AMP (cAMP) upon activation by Gα_s_-coupled G-protein coupled receptors (GPCRs). It localizes to primary cilia in several cell types, including many neuron types in the mammalian brain.

Axoneme: in nonmotile mammalian primary cilia, the axoneme consists of nine microtubule doublets regularly positioned along the circumference of a circle in a so-called ‘9+0’ configuration, and it serves as the structural core of the primary cilium.

BBSome: a complex of eight proteins associated with Bardet–Biedl syndrome (BBS) that are mainly involved in regulating the exit of membrane proteins from the primary cilium, and also the ciliary entry of certain GPCRs by coupling them to the intraflagellar transport (IFT) machinery.

Ectosomes: vesicles released from the plasma membrane of cells. They can contain lipids, proteins, and nucleic acids, and are involved in a variety of cellular processes. Ciliary ectosomes are released from the tip of primary cilia and can be enriched in specific ciliary proteins such as GPCRs. Ectosomes are not to be confused with exosomes, which are small endosomally derived membrane microvesicles.

Intraflagellar transport (IFT): the process by which molecules and proteins are actively transported along the primary cilia axoneme toward the tip by kinesin motor proteins and toward the ciliary base by dynein motor proteins.

Nonsensory neurons: neurons of the central nervous system that are not directly involved in sensing inputs from the environment and that have more typical primary cilia, as opposed to sensory neurons such as photoreceptor neurons, which respond to photons, or olfactory neurons, which respond to olfactory molecules via highly specialized primary cilia.

Primary ciliopathies: a group of genetic disorders caused by defects in the structure or function of primary cilia that affects their signalling role. This diverse group of disorders can affect a wide range of organ systems, including the eyes, kidneys, brain, and skeletal system.

Primary cilia length (PCL): here, we defined PCL as the length of primary cilia from the base to the tip as measured in different studies, largely by fluorescence imaging of primary cilia stained for ciliary proteins or of primary cilia labelled with a reporter gene.

Rho-associated kinase (ROCK): a serine/threonine kinase activated by the small GTPase RhoA. ROCK plays a role in regulating of the actin cytoskeleton and therefore acts on PCL.

Transition zone: a specific subregion of primary cilia that acts as a gate to selectively permit the entrance of proteins, helping make the cilium a separate cellular compartment.
